# Supplementary material for: Institutional factors influencing knowledge production for practice: Evidence from nonprofit studies
Source: PLoS One. 2023 Oct 25;18(10):e0293360. doi: 10.1371/journal.pone.0293360 (PMC10599542; doi:10.1371/journal.pone.0293360)
Supplement: S1 Appendix — (PDF) [file pone.0293360.s001.pdf]

Online Appendix

INSTITUTIONAL FACTORS INFLUENCING KNOWLEDGE PRODUCTION FOR PRACTICE: EVIDENCE FROM NONPROFIT STUDIES

Full article available at https://osf.io/qj85m

A Methods **2**
A.1 Nonprofit scholarship by U.S. universities with an NME focus 2
A.2 Semi-automated approach to coding publications 3
A.2.1 Extract topics and topic keywords 3
A.2.2 Determine practice-oriented topics 4
A.2.3 Code articles using topic keywords 5
A.2.4 Evaluate coding results 5
A.2.5 Remove non-relevant articles 6

B Robustness analysis **6**
B.1 Statistical robustness 6
B.2 Measure robustness: Community needs 7

A Methods

A.1 Nonprofit scholarship by U.S. universities with an NME focus

We followed these steps to compile a dataset of the nonprofit scholarship published by universities with a nonprofit management education (NME) focus.

1. Obtained a list of authors who published at least once in nonprofit journals, treating these researchers as “nonprofit scholars” (Walk & Andersson, 2020). This step generated 17,945 authors from all over the world.
2. These nonprofit scholars may publish articles on nonprofits and philanthropy (or articles that are not relevant to the field) elsewhere. Therefore, we retrieved all the articles published by these authors, generating 307,935 publications.
3. Refined these articles to those with affiliations in the NME dataset (i.e., U.S. universities with an NME focus). This step reduced the literature pool to 101,580 articles.
4. These articles, even though written by nonprofit scholars with affiliations with an NME focus, are still too broad and might be irrelevant to the field. We further restricted the articles to those having at least one of these keywords in their abstracts according to the keyword lists used by numerous studies (Ma & Konrath, 2018; Shier & Handy, 2014; Smith, 2013): “nonprofit,” “non-profit,” “third sector,” “donation,” “donate,” “giving,” “philanthrop,” “prosocial,” “altrui,” “charity,” “charitable,” “volunteering,” “volunteerism,” “NGO,” “nongovernm,” “civil socie.”

The final dataset has the bibliographical details of 6,201 unique articles published between 1951 and 2022, from 245 U.S. universities with an NME focus.

A.2 Semi-automated approach to coding publications

The semi-automated approach identifies three types of information essential in human coding: (1) informative keywords that present the articles' main ideas, (2) the keywords' semantic contexts, and (3) the relevance of keywords and contexts to practice-oriented topics.

A.2.1 Extract topics and topic keywords

For a computer to analyze texts, the texts need to be converted to vectors, a process often referred to as "encoding." There are primarily two approaches to encode texts: the Bag-of-Words approach, which relies on word frequency; and word semantics, which tries to semantically map words in high-dimensional vector spaces (Jurafsky & Martin, 2022; Ma, 2021). We adopted the latter because it is one of the most recent advances in computational linguistics and the basis for many state-of-the-art (SOTA) natural language processing algorithms.

We used the SOTA algorithm devised by Angelov (2020) to extract the topics from the scholarly articles. These topics are represented using keywords. Two technical caveats warrant attention:

1. The number of topics. Running the algorithm with different parameters can generate different numbers of topics, but how many topics make the "correct" number? Defining the "ground truth" of research topics is hardly possible. According to Frumkin (2002, p. 25), it is possible to group the modeled topics into four main categories in order to establish content validity (i.e., service delivery, social entrepreneurship, civic and political engagement, and values and faith). To ensure discriminant validity, the representative keywords within each category should be as far apart as possible in the trained semantic space. For example, if the modeled topics are divided into four categories, the word "political" should be closely associated with one of the four topic vectors, but distinct from the other three. An ideal classification of research topics would produce values such as [0.98, 0.99, 0.92, 0.99]. To evaluate these values, three criteria can be used: 1) the four

numbers should be evenly distributed, which can be assessed using a variant of the Herfindahl–Hirschman index; 2) the average of the four numbers should be high; and 3) as many topics as possible should be retained in order to retain more information and better differentiate articles. The harmonic mean of these three criteria was calculated to determine the optimal number of topics. Based on these strategies, we identified 29 topics from the corpus.

2. The number of keywords. The keywords are not equally helpful, and less informative keywords may introduce noises. The keywords for each topic are ranked according to their centrality to the contents of topics (i.e., the first keyword is more informative than the second keyword in determining what a topic is about; the second is more informative than the third, and so on). The algorithm returns 50 keywords and their centrality values for each topic. This technical caveat is important in informing our analysis, as Appendix A.2.3 elaborates.

A.2.2 Determine practice-oriented topics

It can be hard to draw the line between theoretical and practical scholarship because practitioners and scholars may see the distinction differently. Even among scholars, interpretations can vary because of differences in disciplinary background and research context.

Table A1 lists all the themes found by the authors of the two most-cited articles on this research topic and show how these topics are coded in respective studies (Brudney & Kluesner, 1992; Bushouse & Sowa, 2012). The table shows many inconsistencies, especially for topics concerning the political aspects of nonprofits, government-nonprofit relations, interorganizational relations, and international and macro perspectives. Some similar topics are coded differently in different studies. For example, “government-voluntary sector relations” is labeled as theory-oriented in Brudney and Kluesner (1992), while “government-nonprofit relations” is labeled as practice-oriented in Bushouse and Sowa (2012). These differences mainly reflect the authors’ disciplinary backgrounds and contexts (e.g., sociology and political science versus public

administration and management). Therefore, when coding the extracted topics, we used Table A1 as a reference but also considered the disciplinary background of the keywords.[1]

A.2.3 Code articles using topic keywords

The next step is to calculate the textual similarity between articles' abstracts and topic keywords using the methods applied in Kozlowski et al. (2019), Kusner et al. (2015), Ma (2022), and Ma and Bekkers (2023). The topic with the greatest similarity to an abstract is assigned as the theme of that article. The article is then labeled as practice-oriented or not according to the topic's category.

In a nutshell, we coded the research articles using topic keywords as an instrument. Readers may naturally wonder, since each article is already grouped into one of the topics at the preceding step (i.e., "A.2.2 Determine practice-oriented topics"), why not directly assign the practice label to respective articles? Why do we need to add a step calculating the similarity between topic keywords and research abstracts? There are two principal reasons. 1) As Appendix A.2.1 describes, not all keywords are equally informative, and less informative keywords can introduce errors. Therefore, we only kept keywords with centrality values above the 50th percentile (i.e., the first 25 keywords) at this step. 2) As Appendix A.2.5 describes, some articles may be grouped into non-relevant topics. These topics must be excluded from analysis, and some articles need to be reassigned to nonprofit-relevant topics.

A.2.4 Evaluate coding results

We randomly selected 296 records from the 6,603 total abstracts to manually estimate the quality of the dataset and coding results (confidence level $\approx 95\% \pm 5.6\%$). Among the 296 records, 21.62% of them (64 articles) were not relevant to nonprofit studies. For the 232 articles that were relevant to nonprofits and philanthropy, the consistency of coding between human and algorithm was 84.05% (195 out of 232 records).

A.2.5 Remove non-relevant articles

The dataset contains many non-relevant articles (21.62%, with a confidence level $\approx 95\% \pm 5.6\%$). Although our initial analysis with all topics showed a fair instrumental validity, we could still improve the data quality by removing noisy references.

The 64 non-relevant articles manually checked out primarily include keywords or themes such as "caregiver," "organ donor," and health industries. These articles correspond to the five non-relevant topics extracted in Appendix A.2.1. We can exclude these articles (Table A2) from further analysis.

In total, 387 articles were excluded at this step, and the final dataset had 6,201 articles for further analysis. We randomly selected 117 records from the excluded articles and 163 records from the nonprofit studies-relevant articles to manually validate the quality of the final dataset. Table A3 shows the validation results. The precision is 82.82% ($\frac{TruePositive}{TruePositive+FalsePositive}$, i.e., articles identified as relevant to nonprofit studies really are relevant), and recall is 90.60% ($\frac{TruePositive}{TruePositive+FalseNegative}$, i.e, articles that should be identified as relevant to nonprofit studies are categorized as such).

B Robustness analysis

B.1 Statistical robustness

Table B4 shows the correlation matrix of continuous explanatory variables and suggests that the poverty rate, one of the primary variables of interest, has only weak correlations with other variables. The values of the variance inflation factor of all the explanatory variables is less than 2 ($Mean = 1.47$). Taken together, the risk of multicollinearity is low, and the estimation coefficients should be statistically robust.

B.2 Measure robustness: Community needs

Poverty rate is an intuitive measure of community needs with the assumption that disadvantaged communities should have more social issues that need to be addressed through research. An alternative measure of a community's well-being is median household income (MHI). Unlike the poverty rate, which measures the percentage of individuals in a community living below the poverty line, MHI is a measure of the income of a community's households. It is not affected by changes in the number of individuals in a household, whereas poverty rate is. As a result, MHI may be a more robust measure for getting a complete and accurate picture of a community's well-being, and may be a better indicator of community needs in some cases.

Table B5 shows the results of running the main regressions by replacing poverty rate with MHI. The results are largely consistent with those of the main regressions in Table 6, and the coefficients in Model 6 show more statistical significance. According to the updated estimation using MHI (i.e., Eq. 1), universities with NPS centers will publish $-0.082 \times MHI + 0.12$ more articles on practice-oriented topics than those without.

$$TotArt = \begin{cases} 0.079 \times Grad + (-0.088) \times RU + (-0.035) \times MHI & \text{if } Center = 0 \\ 0.079 \times Grad + (-0.088) \times RU + (-0.117) \times MHI + 0.12 & \text{if } Center = 1 \end{cases} \quad (1)$$

Table A1: Research topics in Brudney and Kluesner (1992) and Bushouse and Sowa (2012)

		Practice	Theory	BK1992	BS2012
Volunteers					
1	Motivation, recognition, retention of volunteers	x		x	
2	Recruitment of volunteers	x		x	
3	Director of volunteer services	x		x	
4	Nontraditional volunteers	x		x	
5	Supervision and management of volunteers	x		x	
6	Human resource management / volunteer administration	x			x
Professionalization					
7	Professional skills	x		x	
8	Information technology	x			x
9	Governance / boards	x			x
10	Organization change and development	x		x	
11	Planning and evaluation of program	x		x	
12	Effectiveness/performance	x			x
13	Financial management	x			x
14	Accountability	x			x
15	Capacity building	x			x
16	Fund development / fundraising / marketing	x			x
17	Substantive areas	x		x	
Political aspects					
18	Political factors / empowerment	x		x	
19	Citizen participation		x	x	
20	Civic engagement/social capital		x		x
21	Advocacy	x			x
22	Citizen participation in self-help, coproduction		x	x	
Government-nonprofit relations					
23	Government-voluntary sector relations		x	x	
24	Government-nonprofit relations	x			x
lnterorganizational relations					
25	lnterorganizational relations		x	x	
26	Interorganizational relationship	x			x
International aspects					
27	International voluntary sector		x	x	
28	International development / microfinance	x			x
Research					
29	Information/literature sources		x	x	
30	Framing research questions		x	x	
31	Methods		x		x
Macro perspectives of nonprofits and philanthropy					
32	Voluntary associations / sector (general)		x	x	
33	Auspices/nature of the sector		x		x
34	Women and voluntary sector		x	x	
35	Structure of voluntary associations		x	x	
36	Philanthropy	x			x
37	Social policy	x			x
38	Faith-based organizations	x			x

Note: BK1992 = Brudney and Kluesner (1992); BS2012 = Bushouse and Sowa (2012). Using exact topic names from original studies. Text in italics indicates major themes and is re-categorized by current study. To read the table: in Line 24, for example, “government-nonprofit relations” is a topic labeled as practice-oriented in Bushouse and Sowa (2012).

Table A2: NON-RELEVANT ARTICLES BY TOPIC

	Top 5 keywords of topic	#Articles	%Articles
0	facility_nursing_chain_ownership_medicare	164	2.48%
1	caregiving_grandparent_caregiver_grandchild_caregive	78	1.18%
2	organ_procurement_consent_transplant_shortage	55	0.83%
3	covid_pandemic_berlin_gruyter_response	50	0.76%
4	stebbins_jurgen_grotz_horton_smith	40	0.61%

Table A3: VALIDATION OF THE CLASSIFICATION OF ARTICLES

		NPS relevant (validated)	
		Yes	No
NPS relevant (predicted)	Yes	135 (TP)	28 (FP)
	No	14 (FN)	103 (TN)

Note: T, F, P, and N represents True, False, Positive, and Negative, respectively. NPS = Nonprofit and philanthropic studies.

Table B4: CORRELATION MATRIX OF CONTINUOUS EXPLANATORY VARIABLES

	P. R.	I. S.	T. A.	Popu.
Poverty rate	1.0			
Inst. size	0.15	1.0		
#Total articles	0.086	0.54	1.0	
Population	−0.011	0.082	0.030	1.0

Table B5: CHECKING ROBUSTNESS: REPLACING POVERTY RATE WITH MEDIAN HOUSEHOLD INCOME

	(1)	(2)	(3)	(4)	(5)	(6)
Program level						
Graduate	0.055*	0.054*	0.060*	0.060*	0.077**	0.079**
	(1.7)	(1.7)	(1.9)	(1.9)	(2.4)	(2.4)
Organization level						
Research univ.			−0.088**	−0.088**	−0.097**	−0.088**
			(−2.3)	(−2.3)	(−2.6)	(−2.4)
NPS center		0.097*	0.10*	0.10*	0.11*	0.12**
		(1.7)	(1.8)	(1.8)	(2.0)	(2.1)
Community level						
MHI					−0.060***	−0.035*
					(−3.1)	(−1.8)
Shifting logics						
Center × MHI						−0.082*
						(−1.9)
Controls						
Inst. size	−0.0024	0.00090	0.022	0.022	0.019	0.021
	(−0.10)	(0.10)	(1.3)	(1.3)	(1.1)	(1.3)
#Total articles	0.97***	0.96***	0.97***	0.97***	0.98***	0.97***
	(21)	(20)	(20)	(20)	(22)	(22)
Population	−0.019	−0.023	−0.021	−0.021	−0.0059	−0.0082
	(−1.4)	(−1.5)	(−1.4)	(−1.4)	(−0.50)	(−0.60)
Observations	220	220	220	220	220	220
Adjusted R^2	0.94	0.94	0.94	0.94	0.94	0.94

Note: DV = number of practice-oriented articles; MHI = Median household income. All continuous variables are normalized using z-score. t statistics in parentheses. * $p < 0.10$, ** $p < 0.05$, *** $p < .01$

References

Angelov, D. (2020). Top2vec: Distributed representations of topics. *arXiv:2008.09470 [cs, stat]*.

Brudney, J. L., & Kluesner, T. M. (1992). Researchers and practitioners in nonprofit organization and voluntary action: Applying research to practice? *Nonprofit and Voluntary Sector Quarterly*, *21*(3), 293–308. https://doi.org/10.1177/089976409202100307

Bushouse, B. K., & Sowa, J. E. (2012). Producing knowledge for practice assessing NVSQ 2000-2010. *Nonprofit and Voluntary Sector Quarterly*, *41*(3), 497–513. https://doi.org/10.1177/0899764011422116

Frumkin, P. (2002). *On being nonprofit: A conceptual and policy primer*. Harvard University Press.

Jurafsky, D., & Martin, J. H. (2022, January 12). *Speech and language processing* (3rd draft).

Kozlowski, A. C., Taddy, M., & Evans, J. A. (2019). The geometry of culture: Analyzing the meanings of class through word embeddings. *American Sociological Review*, *84*(5), 905–949. https://doi.org/10.1177/0003122419877135

Kusner, M., Sun, Y., Kolkin, N., & Weinberger, K. (2015). From word embeddings to document distances. *International Conference on Machine Learning*, 957–966.

Ma, J. (2021). Automated coding using machine learning and remapping the u.s. nonprofit sector: A guide and benchmark. *Nonprofit and Voluntary Sector Quarterly*, *50*(3), 662–687. https://doi.org/10.1177/0899764020968153

Ma, J. (2022). How does an authoritarian state co-opt its social scientists studying civil society? *VOLUNTAS: International Journal of Voluntary and Nonprofit Organizations*. https://doi.org/10.1007/s11266-022-00510-6

Ma, J., & Bekkers, R. (2023). Consensus formation in nonprofit and philanthropic studies: Networks, reputation, and gender. *Nonprofit and Voluntary Sector Quarterly*, 08997640221146948. https://doi.org/10.1177/08997640221146948

Ma, J., & Konrath, S. (2018). A century of nonprofit studies: Scaling the knowledge of the field. *VOLUNTAS: International Journal of Voluntary and Nonprofit Organizations*, *29*(6), 1139–1158. https://doi.org/10.1007/s11266-018-00057-5

Shier, M. L., & Handy, F. (2014). Research trends in nonprofit graduate studies a growing interdisciplinary field. *Nonprofit and Voluntary Sector Quarterly*, *43*(5), 812–831. https://doi.org/10.1177/0899764014548279

Smith, D. H. (2013). Growth of research associations and journals in the emerging discipline of altruistics. *Nonprofit and Voluntary Sector Quarterly*, *42*(4), 638–656. https://doi.org/10.1177/0899764013495979

Walk, M., & Andersson, F. O. (2020). Where do nonprofit and civil society researchers publish? perceptions of nonprofit journal quality. *Journal of Public and Nonprofit Affairs*, *6*(1), 79–95. https://doi.org/10.20899/jpna.6.1.79-95
