# Supplementary material for: Institutional factors influencing knowledge production for practice: Evidence from nonprofit studies
Source: PLoS One. 2023 Oct 25;18(10):e0293360. doi: 10.1371/journal.pone.0293360 (PMC10599542; doi:10.1371/journal.pone.0293360)
Supplement: Attachment — Submitted filename: review_response.pdf [file pone.0293360.s002.pdf]

1. Please ensure that your manuscript meets PLOS ONE's style requirements, including those for file naming.

We used the official LaTeX template provided by PLOS ONE and double checked the Submission Guidelines.

2. Thank you for stating the following financial disclosure. "The project is partly funded or supported by (1) the Academic Development Funds from the RGK Center, (2) a 2021-22 PRI Award from the LBJ School, (3) library resources through the IU Lilly Family School of Philanthropy, and (4) cloud computing resources through the Texas Advanced Computing Center at UT Austin."

Please state what role the funders took in the study. If the funders had no role, please state: "The funders had no role in study design, data collection, and analysis, decision to publish, or preparation of the manuscript." If this statement is not correct you must amend it as needed. Please include this amended Role of Funder statement in your cover letter; we will change the online submission form on your behalf.

We revised the financial disclosure accordingly, please refer to the cover letter for the updated statement (i.e., first page).

3. We note that Figure 4 and Figure C1 in your submission contain [map/satellite] images which may be copyrighted. All PLOS content is published under the Creative Commons Attribution License (CC BY 4.0), which means that the manuscript, images, and Supporting Information files will be freely available online, and any third party is permitted to access, download, copy, distribute, and use these materials in any way, even commercially, with proper attribution. For these reasons, we cannot publish previously copyrighted maps or satellite images created using proprietary data, such as Google software (Google Maps, Street View, and Earth). For more information, see our copyright guidelines: http://journals.plos.org/plosone/s/licenses-and-copyright.

The figures were produced by Tableau Public, a free visualization tool. However, we cannot identify its license conditions. Therefore, we dropped the two figures and added a sentence to redirect readers to the original visualization on the Tableau platform. The sentence reads: “A geovisualization and details of all the 236 U.S. universities with an NME focus can be accessed here: https://jima.me/us_nme.”

4. Small point, there's a typo on page 2, second line "studies" should read "studied"

Thank you for catching this oversight; we fixed the typo.

5. Table 1. Expand Poverty rate to say "Poverty rate at institutional location" You haven't yet introduced how you conceptualize poverty rate when the table is introduced, so it's confusing to the reader who might interpret community-based institutions as increasing the poverty rate.

We understand how the previous content in Table 1 was unclear. In the revised version, we introduced the concept of poverty rate at the institutional location in the section that discusses community-level factors preceding Table 1 (i.e., "initiatives aimed at reducing the local poverty rate of the institution's vicinity, " Lines 114—115). Additionally, as suggested, we modified the language in Table 1 to reflect this change, replacing "poverty rate" with "Poverty rate at institutional location" (pg. 5).

6. The introduction is entirely missing any discussion of implementation science, a field that was likewise developed to bridge the science-practice/practice-science gap. IS principles could further enrich the introduction, particularly discussions about the importance of context, co-creation, team science, etc.

We acknowledge the relevance of implementation science to our manuscript. In this revised version, we incorporated literature on IS and made the following references in the text of the introduction:

> "The gap between theory and practice, itself theoretically a knowledge production problem, becomes an institutional problem in practice. The academic tenure process, funding criteria, and editorial policies largely favor theory-building but discount practical knowledge. This viewpoint has attracted growing attention because it targets the root causes and provides potential routes to change. Take implementation science for example. This research field was initially created to systematize the implementation of evidence-based programs, highlights the importance of institutional contexts that can impede the integration of evidence or practice-based programs or initiatives in organizations [(Fixsen et al. 2005; Fixsen et al, 2015)]. However, merely having these practices available is not enough for widespread adoption and successful implementation. It is crucial to consider the institutional contexts in which these practices are intended to be implemented, taking into account factors such as leadership support, organizational culture, resource availability, workforce capacity, and external influences [(Wandersman et al. 2008)]."

7. In the measures section, it strikes me as a little odd that the authors critiqued the limitations of articles, yet are using them as the key dependent variable. Given this, the authors might explain a bit more why they chose this measure for the dependent variable and if they considered other sources of information that provide practical information to communities.

We appreciate this insight. We expanded our explanation of the rationale behind choosing articles as the primary measure for our dependent variable. While we acknowledge that articles may not capture the entirety of practical information available to communities, we believe they remain an important and widely recognized source of practical knowledge production. In our revised measures section, we specifically explain that we selected articles as our primary measure due to their feasibility and comprehensiveness within the scope of our study.

8. Under explanatory variables (page 8), clarify if the basic county demographics are of the institution or of the subjects of the article.

Likewise, in Table 2, specify that poverty rate by county is where the institution resides, not the study.

Thank you for raising these issues with clarity. We have made the following revisions:

On page 8, we explain that the basic county demographics mentioned in the explanatory variables section refer to the location of each university examined in this study: "basic county demographics pertaining to the location of each university examined in this study, such as total population" (Lines 285—287)

In Table 2, we have also updated the descriptions to reflect the specific focus of each university under examination. The community-level explanation now includes "Poverty rate by county in which universities are located" and "Total population by county in which universities are located." These modifications ensure that the reader understands that the data relates to the specific location of each institution included in the study.

9. Figure 4, it is unclear why University of Hawaii at Manoa was not included due to space. I would recommend including it for completeness.

The revised manuscript now redirects readers to an online visualization dashboard which has comprehensive details. It reads: "A geovisualization and details of all the 236 U.S. universities with an NME focus can be accessed here: https://jima.me/us_nme."

10. I struggled a bit with understanding Table 4. It might be more understandable if the keywords were listed in two tables, one for practice-oriented and one for theory-oriented, and remove the underscores. As it is, it's not clear how to interpret this table, even with the explanation in the text. It's also not entirely clear how the combination of keywords were derived. More information is needed.

Thanks for this suggestion. We removed the underscores and added a note to redirect readers to the technical details that clarify how these keywords are generated (i.e., "Methods for generating these keywords are detailed in S1 Appendix, 'A.2 Semi-automated approach to coding publications.' ").

We kept the two types of keywords in the same table because they are ranked by the number of articles. Therefore, readers can easily compare the most popular topics and tell if they are practice-oriented or not—a primary focus of this study and of a reader's central interest.

11. Table 6. Is there a way to put the odds ratios in the table? It would make understanding the narrative a bit easier to follow. Also, are you sure the Adjusted R2 are the same across all six models? This may be a typo

Thank you very much for this suggestion. Because it is easier to compare the marginal effects of different explanatory variables using standardized coefficients, and it is easier to interpret the practical meanings of these coefficients (e.g., "universities with NPS centers publish 2.68 more practice-oriented articles than those without," Lines 356—357), we reported the standardized coefficients in the main regression table. We also rounded the R2 values to three decimal places.

12. Discussion section. The first sentence in Practical implications of the framework you state "The theoretical framework coherently explains knowledge production for practice in nonprofit studies and has the potential to guide future action." This seems to be a bit of an overreach given variables that were not able to be included in your model. I would recommend a softer frame for the results "The Theoretical framework provides compelling information about factors associated with…"

We appreciate this feedback. We agree that a softer and more nuanced framing of the results would be appropriate. We revised the sentence you pointed out accordingly.

13. Limitations – I think it is important to acknowledge there are likely more factors than the ones able to be distilled in this manuscript that influence the likelihood of practice-based manuscripts.

Thank you for pointing this out. We made improvements to the tone and content of our limitations section. It now includes a new paragraph at the end of the article. It reads:

> "Lastly, while this study offers useful insights into some of the factors that influence the production of practice knowledge, it is crucial to recognize that there are likely additional factors not addressed in the current paper. While the framework presented is grounded in empirical evidence, it should be considered as a preliminary foundation rather than a definitive conclusion. Future endeavors should incorporate a broader range of nuances and complexities when investigating the process of generating practical knowledge."
