# Supplementary material for: Institutional factors influencing knowledge production for practice: Evidence from nonprofit studies
Source: PLoS One. 2023 Oct 25;18(10):e0293360. doi: 10.1371/journal.pone.0293360 (PMC10599542; doi:10.1371/journal.pone.0293360)
Supplement: Attachment — Submitted filename: review_response_r2.doc [file pone.0293360.s003.doc]

Dear PLOS ONE Editors and Reviewers,

We appreciate the valuable comments and thorough reviews on our manuscript titled “Institutional factors influencing knowledge production for practice: Evidence from nonprofit studies.” We carefully considered all feedback and made the requested revisions.

Reviewer #2: Overall, the authors did a laudable job addressing feedback from the prior review. There remain only a couple of small areas to address:

1) Page 7, paragraph starting on line 240, carefully proofread for tense changes

Thanks for pointing this out. We corrected the tense changes for this paragraph. It now reads: “To study the institutional process of knowledge production for practice, the units of analysis in this project were universities. All dependent and independent variables were measures of a university recorded in the NME dataset. To clarify, our study aimed to understand why, *among the U.S. universities with an emphasis on nonprofit management education*, some produced more practical knowledge on nonprofits and philanthropy than others.”

2) Consider ordering Table 3 by % or # of articles

The table is ordered by the total number of articles (i.e., “#Art.” column).

3) Table 5, note if institutional size is inclusive of graduate and undergraduate, or graduate only.

We revised two places to clarify. (1) In Table 2, where the definition of the variable is explained, it reads: “Institution size, ranked from 1 to 5 according to total students enrolled for credit (including both undergraduate and graduate)”. (2) In Table 5, we revised “Inst. size” to “Inst. size (#total students)”.

4) The limitations section was robust and much improved. I think this was implied in the section, but you may want to consider further highlighting that you were only able to look at published manuscripts and did not look at reports or other products that might have been produced that were practice-oriented.

We revised the first sentence of the abstract to emphasize the units of analysis: “This study theorizes and tests an institutional-logics framework to explain why some universities produce more practice-oriented peer-reviewed journal articles than others, using nonprofit studies as an example.”

Best regards,

The Authors
